# Supplementary material for: Understanding of Collective Atom Phase Control in Modified Photon Echoes for a Near-Perfect Storage Time-Extended Quantum Memory
Source: Entropy (Basel). 2020 Aug 15;22(8):900. doi: 10.3390/e22080900 (PMC7517526; doi:10.3390/e22080900)
Supplement: Supplementary file 1 [file entropy-22-00900-s001.pdf]

Supplementary Information
for "Understanding of collective atom phase control in modified photon echoes for a near perfect, storage time extended quantum memory" by

Rahmatullah and B. S. Ham

In Fig. S1(a), the D and R_1 pulses are resonant to the transition $|1\rangle - |2\rangle$. The two counter-propagating C_1 and C_2 control pulses are resonant to the transition $|2\rangle - |3\rangle$. The optical Bloch and the Maxwell-Schrödinger equations are:

$$\frac{\partial}{\partial t}\sigma_{11}(z,t,\Delta) = i\varepsilon_l(z,t)\big(\sigma_{12}(z,t,\Delta) - \sigma_{21}(z,t,\Delta)\big)\,, \tag{S1}$$

$$\frac{\partial}{\partial t}\sigma_{22}(z,t,\Delta) = i\varepsilon_l(z,t)\big(\sigma_{21}(z,t,\Delta) - \sigma_{12}(z,t,\Delta)\big) + i\varepsilon_j\big(\sigma_{23}(z,t,\Delta) - \sigma_{32}(z,t,\Delta)\big)\,, \tag{S2}$$

$$\frac{\partial}{\partial t}\sigma_{33}(z,t,,\Delta) = i\varepsilon_j\big(\sigma_{32}(z,t,\Delta) - \sigma_{23}(z,t,\Delta)\big)\,, \tag{S3}$$

$$\frac{\partial}{\partial t}\sigma_{12}(z,t,\Delta) = i\Delta\sigma_{12}(z,t,\Delta) + i\varepsilon_l(z,t)\big(\sigma_{11}(z,t,\Delta) - \sigma_{22}(z,t,\Delta)\big) + i\varepsilon_j(z,t)\sigma_{13}(z,t,\Delta), \tag{S4}$$

$$\frac{\partial}{\partial t}\sigma_{32}(z,t,\Delta) = i\Delta\sigma_{32}(z,t,\Delta) + i\varepsilon_j(z,t)\big(\sigma_{33}(z,t,\Delta) - \sigma_{22}(z,t,\Delta)\big) + i\varepsilon_l(z,t)\sigma_{31}(z,t,\Delta), \tag{S5}$$

$$\frac{\partial}{\partial t}\sigma_{13}(z,t,\Delta) = i\varepsilon_j(z,t)\sigma_{12}(z,t,\Delta) + i\varepsilon_l\sigma_{23}(z,t,\Delta)\,, \tag{S6}$$

$$\frac{\partial}{\partial z}\varepsilon_{l(j)}(z,t) = \frac{i\alpha}{2\pi}\int_{-\infty}^{\infty}\sigma_{12}(z,t,\Delta)d\Delta, \tag{S7}$$

where, l= D or R_1 and j= C_1 or C_2. The $\varepsilon_{l(j)}$ is the optical field, α is the optical depth parameter and $\Delta = \omega_{12} - \omega_l = \omega_{23} - \omega_j$ is the detuning of the atom. For the controlled single rephasing photon echo scheme without R_2, the controlled coherence conversion is studied below as discussed in numerically in ref. [18] and experimentally in ref. [19]. The pulse sequence is shown in Fig. S1(b).

I. D-pulse

A weak D-pulse propagates through the medium along z-direction. We assume that D-pulse is much weaker than the π-pulse and neglect population change by D: $\sigma_{11}(z,t,\Delta) = 1$. The resultant Maxwell-Bloch equations by D are obtained by putting $\varepsilon_l = \varepsilon_{\mathrm{D}}$ and $\varepsilon_j = 0$ in equations (S4) and (S7)

$$\frac{\partial}{\partial t}\sigma_{12}(z,t,\Delta) = i\Delta\sigma_{12}(z,t,\Delta) + i\varepsilon_{\mathrm{D}}(z,t)\,, \tag{S8}$$

$$\frac{\partial}{\partial z}\varepsilon_{\mathrm{D}}(z,t) = \frac{i\alpha}{2\pi}\int_{-\infty}^{\infty}\sigma_{12}(z,t,\Delta)d\Delta. \tag{S9}$$

The equation (S8) is the first order linear differential equation. By applying integrating factor $e^{-i\Delta t}$, the solution is obtained as:

$$\sigma_{12}(z,t,\Delta) = \sigma_{12}(z,-\infty,\Delta) + i\int_{-\infty}^{t}\varepsilon_D(z,t')e^{i\Delta(t-t')}, \tag{S10}$$

By setting the initial atomic coherence zero, $\sigma_{12}(z,-\infty,\Delta) = 0$,

$$\sigma_{12}(z,t,\Delta) = i\int_{-\infty}^{t}\varepsilon_D(z,t')e^{i\Delta(t-t')}\,dt'. \tag{S11}$$

Here, the coherence is positive (absorptive), so does the echo. It should be noted that $\sigma_{12}(z,t,\Delta) = -\rho_{12}(z,t,\Delta)$, where $\rho_{12}(z,t,\Delta)$ is the density matrix element. Taking the Fourier transform of equation (S11) and substituting into the Fourier version of (S9), we obtain:

$$\frac{\partial}{\partial z}\varepsilon_{\mathrm{D}}(z,t) = -\frac{\alpha}{2\pi}\int_{-\infty}^{\infty}\varepsilon_{\mathrm{D}}(z,\omega)\left[\frac{1}{i(\omega-\Delta)}+\pi(\omega-\Delta)\right]d\Delta = -\frac{\alpha}{2}\varepsilon_{\mathrm{D}}(z,\omega). \quad \text{(S12)}$$

The solution of equation (S12) in time domain is:

$$\varepsilon_{\mathrm{D}}(z,t) = e^{-\frac{\alpha z}{2}}\varepsilon_{\mathrm{D}}(0,t). \quad \text{(S13)}$$

The D-pulse exponentially decays as it propagates through the medium.

II. $\mathrm{R_1}$-pulse

To retrieve D, we apply a π $\mathrm{R_1}$-pulse in delay time T after the D-pulse. By the $\mathrm{R_1}$-pulse the atoms are excited ($\sigma_{22}(z,t,\Delta) = 1$), and the corresponding equations of motion are:

$$\frac{\partial}{\partial t}\sigma_{12}(z,t,\Delta) = i\Delta\sigma_{12}(z,t,\Delta) - i\varepsilon_{\mathrm{R_1}}(z,t)\,, \quad \text{(S14)}$$

$$\frac{\partial}{\partial z}\varepsilon_{\mathrm{R_1}}(z,t) = \frac{i\alpha}{2\pi}\int_{-\infty}^{\infty}\sigma_{12}(z,t,\Delta)d\Delta. \quad \text{(S15)}$$

The equations (S14) and (S15) are obtained by substituting $\varepsilon_l = \varepsilon_{\mathrm{R_1}}$ and $\varepsilon_j = 0$ into equations (S4) and (S7). The solution of equation (S14) yields:

$$\sigma_{12}(z,t,\Delta) = e^{i\Delta(t-t_{\mathrm{R_1}})}\sigma_{12}(z,t_{\mathrm{R_1}},\Delta) - i\int_{t_{\mathrm{R_1}}}^{t}\varepsilon_{\mathrm{R_1}}(z,\acute{t})e^{i\Delta(t-\acute{t})}\,d\acute{t}. \quad \text{(S16)}$$

The $\mathrm{R_1}$-pulse results in a phase conjugate of the D-excited coherence, so the coherence at $t = t_{\mathrm{R_1}}$ is equal to the conjugate of equation (S11):

$$\sigma_{12}(z,t,\Delta) = -\,i\,e^{-i\Delta(2t_{\mathrm{R_1}}-t)}\int_{-\infty}^{\infty}\varepsilon_{\mathrm{D}}^{\dagger}(z,\acute{t})e^{i\Delta\acute{t}}\,d\acute{t} - i\int_{t_{\mathrm{R_1}}}^{t}\varepsilon_{\mathrm{R_1}}(z,\acute{t})e^{i\Delta(t-\acute{t})}\,d\acute{t}. \quad \text{(S17)}$$

The negative sign in equation (S17) shows the emissive coherence of the photon echo. With some mathematical calculations, we obtain the following equation in a frequency domain:

$$\sigma_{12}(z,\omega,\Delta) = -\,i\,e^{-2i\Delta t_{\mathrm{R_1}}}2\pi\delta(\omega-\Delta)\int_{-\infty}^{\infty}\varepsilon_{\mathrm{D}}^{\dagger}(z,\acute{t})e^{i\Delta\acute{t}}d\acute{t} - i\varepsilon_{\mathrm{R_1}}(z,\omega)\left[\frac{1}{i(\omega-\Delta)}+\pi\delta(\omega-\Delta)\right]. \quad \text{(S18)}$$

By taking the Fourier transform of equation (S15) and substituting equation (S18), we get:

$$\varepsilon_{\mathrm{R_1}}(z,t) = \varepsilon_{\mathrm{R_1}}(0,t)e^{\frac{\alpha z}{2}} + 2\sinh\left(\frac{\alpha z}{2}\right)\varepsilon_{\mathrm{D}}^{\dagger}(0,2t_{\mathrm{R_1}}-t). \quad \text{(S19)}$$

The echo is emitted at $t = 2t_{\mathrm{R_1}} - t_{\mathrm{D}}$. The efficiency of the echo is $4\sinh^2\left(\frac{\alpha z}{2}\right)$, which is greater than unity for a large optical depth due to the stimulated emission in the inverted medium [1,2].

III. C_1 and C_2 -pulses

The function of C_1–pulse is to convert the optical coherence $\sigma_{12}(z,t,\varDelta)$ into spin coherence $\sigma_{13}(z,t,\varDelta)$. The coherence at $t_{C_1}$is given by:

$$\sigma_{12}(z,t,\varDelta) = -\,i\,e^{-i\Delta(2t_{R_1}-t_{C_1})}\int_{-\infty}^{\infty}\varepsilon_D^{\dagger}(z,\acute{t})e^{i\Delta\acute{t}}\,d\acute{t}. \tag{S20}$$

Here, we consider the first term of equation (S17) related to the evolution of the coherences excited by the D-pulse. The optical coherence is transferred to spin coherence via relation; $\sigma_{12}(z,t,\varDelta) = \cos\left(\frac{\pi}{2}\right)\sigma_{12}\left(z,t_{C_1},\varDelta\right) = 0$ and $\sigma_{13}(z,t,\varDelta) = e^{i\pi/2}\sigma_{12}\left(z,t_{C_1},\varDelta\right)$ [17,18]. The π C_1 –pulse resulting additional $\pi/2$ phase shift, leading to the freezing of the optical coherence. The C_2 –pulse transfers back the spin coherence to optical coherence. By setting $\varepsilon_l = 0$ and $\sigma_{13}(z,t,,\varDelta) = 0$ in equation (S4), we get:

$$\frac{\partial}{\partial t}\sigma_{12}(z,t,\varDelta) = i\varDelta\sigma_{12}(z,t,\varDelta). \tag{S21}$$

The solution of (S21) is:

$$\sigma_{12}(z,t,\varDelta) = \sigma_{12}\left(z,t_{C_2},\varDelta\right)e^{i\Delta(t-t_{C_2})}, \tag{S22}$$

The π C_2 –pulse re-swaps the coherences with another $\pi/2$ phase shift.

$$\sigma_{12}\left(z,t_{C_2},\varDelta\right) = e^{i\pi/2}\sigma_{13}(z,t,\varDelta) = e^{i\pi}\sigma_{12}\left(z,t_{C_1},\varDelta\right) = i\,e^{-i\Delta(2t_{R_1}-t_{C_1})}\int_{-\infty}^{\infty}\varepsilon_D^{\dagger}(z,\acute{t})e^{i\Delta\acute{t}}\,d\acute{t}. \tag{S23}$$

Substituting equation (S23) into (S22), we obtain:

$$\sigma_{12}(z,\mathrm{t},\varDelta) = i\,e^{-i\Delta(t_{C_2}-t_{C_1}+2t_{R_1}-t)}\int_{-\infty}^{\infty}\varepsilon_D^{\dagger}(z,\acute{t})e^{i\Delta\acute{t}}\,d\acute{t}. \tag{S24}$$

Here the final atomic coherence is positive. Thus, the echo becomes absorptive and cannot be radiated from the medium [18]. If the area of the C_2 is 2π, it brings the atomic coherence halted ($\sigma_{12}(z,t) = 0$) again transferring it to the spin state $|3\rangle$. For 3π-C_2, the atoms are returned in the excited state $|2\rangle$, and $\sigma_{12}\left(z,t_{C_2}t_{C_2},\varDelta\right) = -\sigma_{12}\left(z,t_{C_2}t_{C_2},\varDelta\right)$ in equation (S24), where the echo becomes emissive. Therefore, π-3π control pulse sequence is valid for a single rephasing scheme as shown in ref. [18]. Thus, the controlled AFC echoes with the π-π control pulse sequence [8,9] results in an absorptive echo [3]. The observation of the controlled AFC is due to coherence leakage by spatial Gaussian distribution of laser light [20].

References

[1] M. Azadeh, C. Sjaarda Cornish, W. R. Babbitt, and L. Tsang, Phys. Rev. A **57**, 4662 (1998).
[2] J. Ruggiero, J.-L. Le Gouët, C. Simon, and T. Chanelière, Phys. Rev. A **79**, 053851 (2009).
[3] B. S. Ham, J. Opt. Soc. Am. B **28**, 775 (2011).

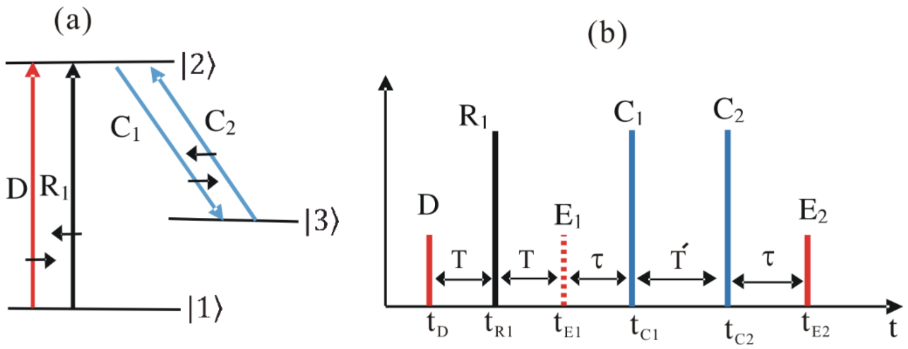

Fig. S1. (a) Schematic single rephasing echoes in a three-level system. (b) Pulse sequence for (a). tj is the arrival time of pulse j
